# Interrupted Time Series of User‐centered Clinical Decision Support Implementation for Emergency Department–initiated Buprenorphine for Opioid Use Disorder

**DOI:** 10.1111/acem.14002

**Published:** 2020-05-19

**Authors:** Wesley C. Holland, Bidisha Nath, Fangyong Li, Kaitlin Maciejewski, Hyung Paek, James Dziura, Haseena Rajeevan, Charles C. Lu, Liliya Katsovich, Gail D'Onofrio, Edward R. Melnick

**Affiliations:** ^1^ Yale School of Medicine New Haven CT; ^2^ Department of Emergency Medicine New Haven CT; ^3^ Yale Center for Analytical Sciences New Haven CT; ^4^ Information Technology Services Yale New Haven Health New Haven CT

## Abstract

**Objectives:**

Adoption of emergency department (ED) initiation of buprenorphine (BUP) for opioid use disorder (OUD) into routine emergency care has been slow, partly due to clinicians’ unfamiliarity with this practice and perceptions that it is complicated and time‐consuming. To address these barriers and guide emergency clinicians through the process of BUP initiation, we implemented a user‐centered computerized clinical decision support system (CDS). This study was conducted to assess the feasibility of implementation and to evaluate the preliminary efficacy of the intervention to increase the rate of ED‐initiated BUP.

**Methods:**

An interrupted time series study was conducted in an urban, academic ED from April 2018 to February 2019 (preimplementation phase), March 2019 to August 2019 (implementation phase), and September 2019 to December 2019 (maintenance phase) to study the effect of the intervention on adult ED patients identified by a validated electronic health record (EHR)‐based computable phenotype consisting of structured data consistent with potential cases of OUD who would benefit from BUP treatment. The intervention offers flexible CDS for identification of OUD, assessment of opioid withdrawal, and motivation of readiness to start treatment and automates EHR activities related to ED initiation of BUP (including documentation, orders, prescribing, and referral). The primary outcome was the rate of ED‐initiated BUP. Secondary outcomes were launch of the intervention, prescription for naloxone at ED discharge, and referral for ongoing addiction treatment.

**Results:**

Of the 141,041 unique patients presenting to the ED over the preimplementation and implementation phases (i.e., the phases used in primary analysis), 906 (574 preimplementation and 332 implementation) met OUD phenotype and inclusion criteria. The rate of BUP initiation increased from 3.5% (20/574) in the preimplementation phase to 6.6% (22/332) in the implementation phase (p = 0.03). After the temporal trend of the number of physician's with X‐waiver training and other covariates were adjusted for, the relative risk of BUP initiation rate was 2.73 (95% confidence interval [CI] = 0.62 to 12.0, p = 0.18). Similarly, the number of unique attendings who initiated BUP increased modestly 7/53 (13.0%) to 13/57 (22.8%, p = 0.10) after offering just‐in‐time training during the implementation period. The rate of naloxone prescribed at discharge also increased (6.5% preimplementation and 11.5% implementation; p < 0.01). The intervention received a system usability scale score of 82.0 (95% CI = 76.7 to 87.2).

**Conclusion:**

Implementation of user‐centered CDS at a single ED was feasible, acceptable, and associated with increased rates of ED‐initiated BUP and naloxone prescribing in patients with OUD and a doubling of the number of unique physicians adopting the practice. We have implemented this intervention across several health systems in an ongoing trial to assess its effectiveness, scalability, and generalizability.

An estimated 2.1 million people nationally suffer from opioid use disorder (OUD), contributing to nearly 50,000 overdose deaths each year.^1^, ^2^ With 605,000 opioid‐related emergency department (ED) visits in 2011 and a 30% increase in visits for opioid overdose‐related visits between 2016 and 2017, the ED is a major and increasingly utilized setting for OUD treatment.^3^, ^4^ People with OUD not only seek emergency care in high‐acuity situations like overdose and withdrawal, but also for comorbid or general health issues.^5^ Given the stigma associated with OUD, the ED may serve as the primary access to health care for this vulnerable patient population.^5^, ^6^ Thus, the ED provides a unique opportunity to initiate appropriate treatment for OUD.^7^, ^8^, ^9^

Opioid agonist medications, such as buprenorphine/naloxone (BUP) and methadone, are the current standard of treatment for OUD and have been shown to reduce withdrawal symptoms, craving, relapse, overdose, and mortality (all cause and opioid related).^10^, ^11^, ^12^ A 2015 randomized clinical trial involving 329 ED patients with OUD demonstrated that BUP can be safely initiated in the ED and demonstrated that patients receiving BUP in the ED were twice as likely to remain engaged in formal addiction treatment at 1 month (78% vs. 37%, p < 0.001).^13^ Despite this evidence, BUP initiation in the ED has been slow to be adopted into routine emergency care to replace the current standard of care that historically has included symptomatic treatment for opioid withdrawal symptoms and referral for addiction treatment without addressing the underlying disorder.^5^, ^7^, ^9^ Furthermore, the rate of naloxone prescription upon ED discharge following nonfatal overdose remains low even as an evidence‐based practice known to decrease mortality and risk of future overdose.^11^, ^14^ Just as the ED is a unique setting to increase rates of BUP initiation, it is also an opportunity to implement other harm reduction strategies such as naloxone prescribing.^15^

Numerous patient‐side barriers currently limit the adoption of BUP initiation, including confusion and cultural stigma surrounding medication therapy for OUD and patient perceptions that such treatment is harmful, inferior to detoxification, and even incompatible with being truly “drug‐free.”^16^, ^17^, ^18^ The lack of adoption of ED initiation of BUP into routine emergency care has been attributed to emergency clinicians' lack of training in addiction treatment and perception that BUP initiation is unfamiliar, complicated, and time‐consuming.^5^, ^19^ One potential solution previously shown to provide effective guidance for drug therapy is clinical decision support (CDS), computerized systems that provide patient‐specific guidance.^20^, ^21^, ^22^

To address these barriers to implementation and to simplify the practice of ED‐initiated BUP, we developed a user‐centered CDS called EMBED (EMergency department‐initiated BuprenorphinE for opioid use Disorder).^23^ To assess the feasibility of implementation and to evaluate the preliminary efficacy of the intervention to increase adoption of ED initiation of BUP, it was implemented in a single ED as the intervention in an interrupted time series study. The lessons learned from this study, particularly the qualitative feedback regarding intervention improvement, can be applied to a subsequent pragmatic group randomized trial involving 20 EDs across five health care systems. This multisystem pragmatic trial will determine the effectiveness of the EMBED intervention on the adoption of ED‐initiated BUP for OUD.^24^

## Methods

### Study Design and Setting

A single‐site time series study evaluating the preliminary efficacy of the EMBED intervention was conducted during April 2018 to December 2019 in an urban, academic Level I trauma center ED with 103,000 annual patient visits. The time series was divided into three phases for analysis: 1) preimplementation phase (April 2018–February 2019), 2) implementation phase (March 2019–August 2019), and 3) maintenance phase (September 2019–December 2019). The phased rollout of the CDS intervention began with a soft go‐live in mid‐January 2019. Users were then made aware of the CDS's availability when full functionality was achieved in early March 2019. Provider feedback on the CDS was collected in the implementation phase to assist with planning the subsequent trial. The study protocol was reviewed and approved by our institutional review board (Protocol 2000022749).

### Subjects

**Figure 1:**
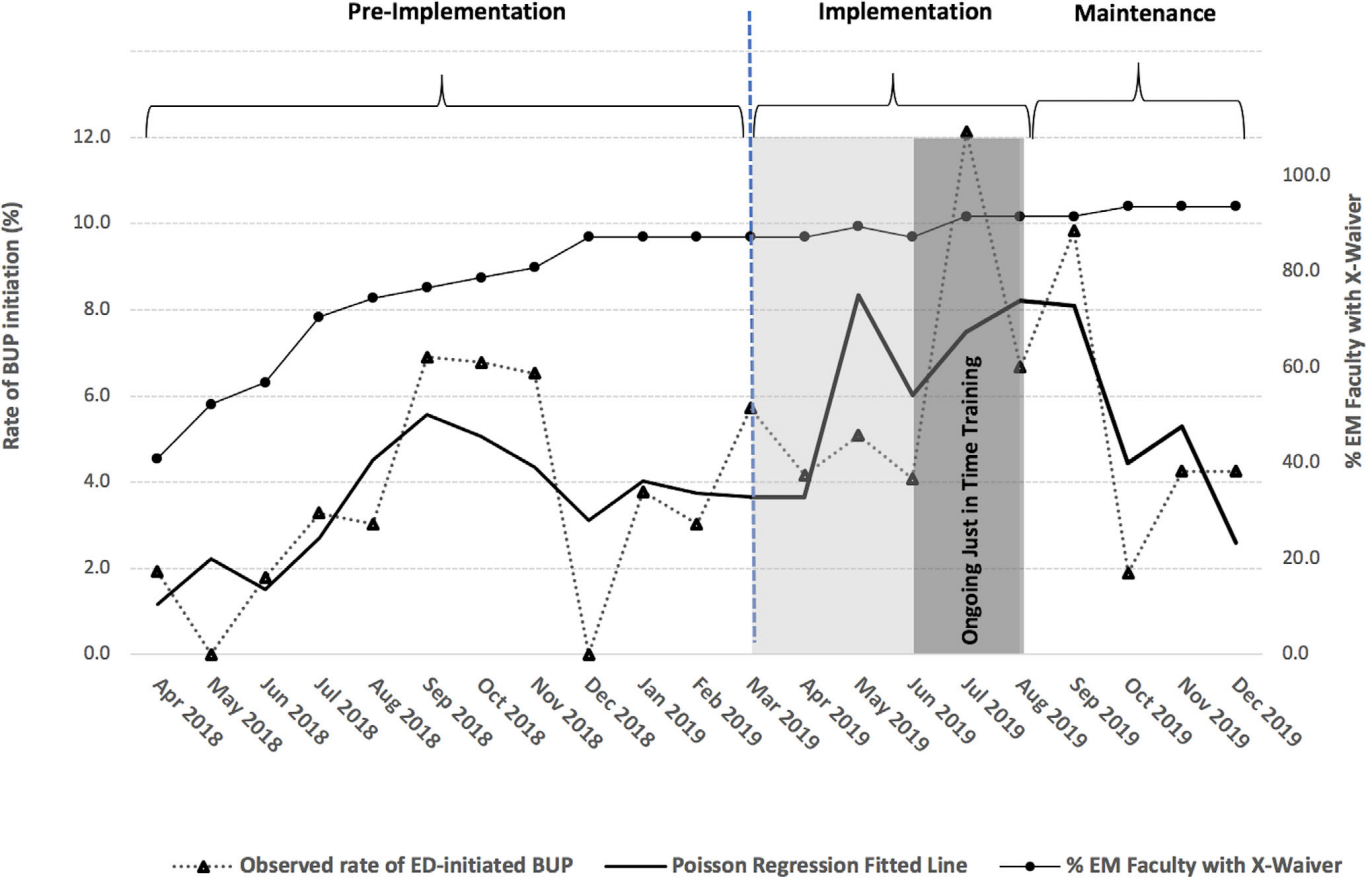
Unadjusted patient and physician outcomes in the preimplementation, implementation, and maintenance periods. BUP = buprenorphine.

Eligible patients were adult ED patients meeting the criteria of a computable phenotype derived from electronic health record (EHR) data that was developed to capture ED patients likely to have OUD and not actively on medication for OUD (MOUD, i.e., methadone, BUP, or naltrexone).^25^ The phenotype is comprised of two algorithms: one based on clinician and billing codes (Algorithm 1) and the other based on structured EHR data of the chief complaint (Algorithm 2). Additionally, the phenotype excludes patients who are admitted to the hospital or pregnant. In this way, the phenotype was designed to maximize specificity in identification of patients eligible for BUP initiation. Development, internal validation, and external validation of the phenotype occurred across two large health care systems containing 13 EDs. The phenotype has an externally validated positive predictive value of 0.95 and a negative predictive value of 0.92.^25^ A waiver of informed consent was obtained given that data were only collected retrospectively and did not involve patient interaction or identifiable information. Regarding clinician subject inclusion criteria, attending ED physicians practicing at the intervention site who cared for the phenotype‐positive patients were eligible for inclusion. Given the additional burden of the consent process and to ensure the validity of the intervention's efficacy on changing routine care, clinician demographic information was not collected.^24^, ^26^ Therefore, all patient and physician identifiers were removed from EHR data by an honest broker and not shared with the investigative team. As a result of this deidentification process, it was not possible to match physician study data to our faculty roster to determine which physicians had an X‐waiver to prescribe BUP. Therefore, a separate emergency medicine faculty roster was used to determine the proportion of physicians with an X‐waiver (Figure 1).

### Intervention

**Figure 2:**
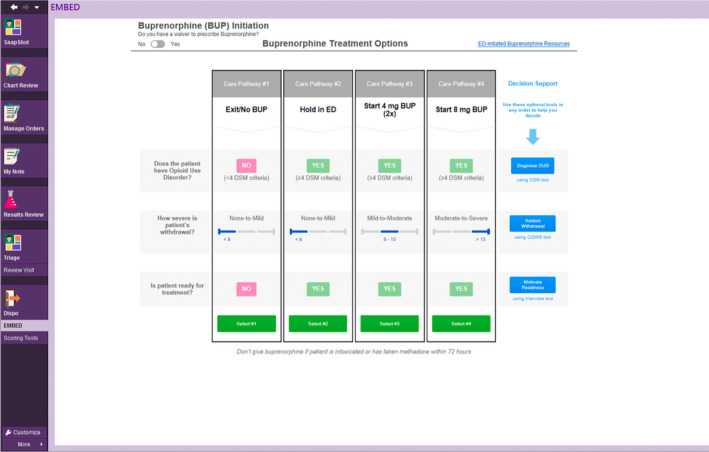
Screenshot of EMBED with care pathways welcome screen within EHR workflow. EHR = electronic health record; BUP = buprenorphine.

The study intervention included an integrated Web application for decision support and automation of EHR workflow that streamlines the practice of initiating BUP in the ED (Figure 2). Full details of the intervention's design and IT integration have been previously reported.^23^, ^27^, ^28^ Briefly, the intervention is launched at the clinician's discretion for patients who they suspect may have OUD by clicking the “EMBED” button on the navigation bar of a patient's chart (the phenotype did not flag or alert clinicians of OUD cases). This opens a Web application within the EHR that offers three optional decision support tools to inform clinicians' selection of the appropriate care pathway through the diagnosis of OUD, assessment of withdrawal severity, and motivation of patient readiness to start treatment for OUD. The clinician can choose to use all, some, or none of these tools. Once the care pathway is selected, the Web application automates a series of EHR activities specific to that pathway including appropriate orders, prescriptions, documentation, discharge instructions, and referral to a community provider of MOUD. Early audits in the implementation period identified low intervention use despite targeted e‐mail communication and group lectures.

To enhance use, midway through the implementation period, 5‐minute one‐on‐one tutorials were performed by author WCH to provide just‐in‐time training as an additional academic detailing component of the intervention. Compared to CDS alone, academic detailing combined with CDS has been shown to increase use when introducing a new CDS.^29^ Used in both industry and medicine and usually occurring in a real‐time work setting, just‐in‐time training is an approach that involves presenting relevant information for immediate application.^30^, ^31^ Clinicians were compensated with a $10 Starbucks gift card for their participation in the tutorial and a brief follow‐up interview (Data Supplement S1, Appendix S1, available as supporting information in the online version of this paper, which is available at http://onlinelibrary.wiley.com/doi/10.1111/acem.14002/full). A convenience sample of physicians completing the tutorial was utilized to gather qualitative feedback via semistructured interview questions. A formal qualitative analysis was not performed, but feedback on the CDS obtained through these interviews was synthesized and categorized according to common, recurring themes as displayed in Data Supplement S1, Appendix S2.

### Outcomes

The primary outcome of this study was BUP initiation rate in the ED, defined as whether or not an eligible patient was administered BUP in the ED and/or prescribed BUP on discharge from the ED. Secondary outcomes to evaluate preliminary efficacy of the intervention's implementation included the attending physician adoption rate of the practice of ED initiation of BUP at least once in the study phase as well as the following patient‐level rates in the cascade of care for treatment among eligible patients:^27^, ^32^ 1) launch of the intervention, 2) referral to follow‐up for ongoing MOUD treatment as documented in the EHR, and 3) prescription for naloxone at ED discharge.

The RE‐AIM (reach, effectiveness, adoption, implementation, and maintenance) framework was also used to evaluate the success of the intervention.^33^, ^34^, ^35^The *reach *of the intervention—the proportion of the target population that participated in the intervention—was the proportion of unique attendings who ever launched the intervention. *Effectiveness* was assessed based on the rate of BUP initiation in the ED during the implementation period (the primary outcome). *Adoption* was based on the proportion of unique attendings who initiated BUP, and *Implementation*—the extent to which the intervention is implemented as intended—was the proportion of phenotype‐positive patients for whom the intervention was launched. *Maintenance* was the rate of BUP initiation in the ED after training ended (September–December 2019).

### Data Collection

Quantitative outcome data from the three study periods were extracted from the study site's EHR database using structured query language (SQL). The SQL query (Data Supplement S2, Appendix S3) included all data elements specified in the master data dictionary created for the subsequent trial (Data Supplement S2, Appendix S4).

Additional qualitative and quantitative data regarding clinician perceptions of barriers to implementation and usability of the intervention were collected via one‐on‐one interviews with clinicians (attendings, residents, APRNs, and PAs) performed by author WCH in the ED. The interview consisted of three parts: 1) system usability scale (SUS), 2) net promoter score (NPS), and 3) three open‐ended questions focusing on barriers to implementation and ways to address them (Data Supplement S1, Appendix S1). The SUS is a 10‐item usability assessment that is widely used and considered the industry standard for rapid assessment of health IT usability; a score of >70 is considered “acceptable.”^36^, ^37^ NPS is a single‐item measure of how likely an individual would be to recommend a product, company, or service to a friend or colleague.^38^

### Data Analysis

Patient characteristics were summarized as means and standard deviations (SDs) or frequencies and percentages as appropriate for the preimplementation and implementation periods. For patients with multiple visits, only the first visit was used to analyze patient‐level outcomes. However, analysis of physician‐level outcomes (i.e., BUP initiation, launching the intervention) included multiple visits made by a single patient. T‐tests and chi‐square or Fisher's exact tests were used to make unadjusted comparisons of demographics, primary outcome rates of BUP initiation and secondary patient outcomes between periods. McNemar's test was used to compare the number of unique attendings who initiated BUP who were present during both the preimplementation and the implementation phases, while the generalized estimating equation method was used as a supportive analysis of all attendings (i.e., including those not present in both time periods). Following unadjusted analysis, a multivariate logistic regression model was used to adjust for age, race, sex, the number of waivered physicians, naloxone prescription within the past 24 months, and OUD diagnosis on problems list. To further contrast BUP initiation between study phases while “detrending” the time effect, Poisson regression was utilized for the interrupted time series adjusted analyses. For this analysis, an offset was used for the monthly volume of patients presenting to the ED with OUD. Relative risk and 95% confidence interval (CI) are reported, with values above 1 corresponding to greater relative rates of ED BUP initiation. Physician X‐waiver status was used as a covariate in this analysis. All analyses were performed using SAS 9.4. Statistical significance was set as p < 0.05, two‐sided.

## Results

### Subject Characteristics

Of the 141,014 total ED visits during the two study phases used for analysis of main outcomes, 906 (574 preimplementation and 332 implementation) met inclusion criteria for analysis. Of these 906 OUD phenotype–positive visits, 98 patients in total (11%) had more than one ED visit, including a maximum of four visits (of which four patients had). Across these two phases, patients had a mean age of 39.9 years, 31.2% were female, 71.7% were white, and 73.4% had Medicaid insurance (Table 1).

**Table 1:** Subject Characteristics

Characteristic	Preimplementation (*n* = 574)	Implementation (*n* = 332)	p‐value
Age (year)	40.2 (±12.6)	39.4 (±12.1)	0.34
Sex, *n* (% female)	172 (30.0)	111 (33.4)	0.28
Race			0.001
Black or African American	90 (15.7)	43 (13.0)	
White or Caucasian	425 (74.0)	225 (67.8)	
Asian, American Indian, or Alaska	2 (0.3)	6 (1.8)	
Other	57 (9.9)	58 (17.5)	
Ethnicity			0.32
Hispanic or Latino	91 (15.9)	65 (19.6)	
Non‐Hispanic	481 (83.8)	264 (79.5)	
Other	2 (0.4)	3 (0.9)	
Insurance information			0.56
BCBS or commercial	32 (5.6)	22 (6.6)	
Managed care	35 (6.1)	15 (4.5)	
Medicaid	426 (74.2)	239 (72.0)	
Medicare	55 (9.6)	35 (10.5)	
Other	26 (4.5)	21 (6.3)	
Phenotype			0.57
Algorithm 1	368 (64.1)	219 (66.0)	
Algorithm 2	206 (35.9)	113 (34.0)	
Naloxone prescribed during encounter as inpatient medication	24 (4.2)	17 (5.1)	0.51
Prescribed naloxone within past 24 months	30 (5.2)	27 (8.1)	0.08
OUD diagnosis on problems list at time of encounter	105 (18.3)	83 (25.0)	0.02
Urine drug screen	88 (15.3)	56 (16.9)	0.54
Positive for opioids	52 (59.1)	26 (46.4)	0.14
Positive for oxycodone	9 (10.2)	7 (12.5)	0.67

Data are reported as mean (±SD) or *n* (%).

BCBS = Blue Cross Blue Shield; OUD = opioid use disorder.

### Main Results

#### Primary Outcome

The rate of BUP initiation (i.e., BUP administered in the ED and/or prescribed on discharge) was 3.5% (20/574) in the preimplementation phase and 6.6% (22/332) in the implementation phase (p = 0.03; Table 2, Figure 1). Compared to the beginning of the implementation period, the rate of BUP initiation was higher after just‐in‐time training was offered as an additional component of the intervention (7.9% vs. 4.9%, p = 0.28). After adjusting for age, race, sex, number of waivered physicians, naloxone prescription within the past 24 months, and OUD diagnosis on problems list, the odds of ED‐initiated BUP was 1.83 in the implementation phase compared to preimplementation (95% CI = 1.03 to 3.25). BUP initiation relative risk adjusting for the same covariates as well as the time trend with Poisson regression were 2.73 (95% CI = 0.62 to 11.99, p = 0.18) for implementation versus preimplementation phase. Of note, the significant difference between BUP initiation rates in the implementation and preimplementation phase persisted with adjustment for time trend, age, race, sex, naloxone prescription within the past 24 months, and OUD diagnosis on problems list and was only lost after adjusting for X‐waiver status over time.

**Table 2:** Outcomes

Outcome	Preimplementation *n* = 574	Implementation *n* = 332	p‐value
Primary outcome			
BUP administered in ED or prescribed on discharge	20 (3.5)	22 (6.6)	0.03
Secondary outcomes			
Prescription for naloxone at ED discharge	37 (6.5)	38 (11.5)	0.009
Receipt of discharge instruction on opioid use, overdose education, naloxone education, and BUP education	218 (38.0)	115 (34.6)	0.32
Referral for ongoing MOUD	97 (16.9)	60 (18.1)	0.65
Number of unique attendings present in both phases who initiated BUP	14/58 (24.1)	16/58 (27.6)	0.65*
Rate of physician intervention launched per 100 phenotype‐positive patients		7.3 (4.8–9.8)	—

Data are reported as *n* (%) or mean (95% CI).

BUP = buprenorphine; MOUD = medication for opioid use disorder; OUD = opioid use disorder.

* McNemar's test.

#### Secondary Outcomes

More subjects received a prescription for naloxone at discharge from the ED in the implementation period (6.5% vs. 11.5%, p < 0.01, Table 2). The rate of referral for ongoing MOUD treatment was 16.9% preimplementation and 18.1% in the implementation phase (p = 0.65).

The number of unique attendings who were present in both study phases (inclusive of all physician participants, not just faculty on the roster used to determine X‐waiver status) who initiated BUP did not change significantly from 14/58 (24.1%) in the preimplementation phase to 16/58 (27.6%) in the implementation phase (p = 0.65). After just‐in‐time training was added for the second half of the implementation phase, the number of unique attendings who initiated BUP increased from 7/53 (13.0%) to 13/57 (22.8%, p = 0.10). The addition of just‐in‐time training was also associated with an increase in the proportion of unique attendings who launched the intervention in the implementation period (7/53, 13.0% vs. 15/57, 25.9%; p = 0.07).

Evaluation of the intervention's implementation using the RE‐AIM framework (Table 3) shows that it reached 44% of the target population (unique ED attending physicians who launched the intervention at least once), resulting in 32.3% of attendings adopting the practice of ED initiation of BUP. The rate of BUP initiation in the ED after training ended in the maintenance phase was 5.5% and associated with a decline in BUP initiation rates over time as displayed in Figure 1 (adjusted time trend of BUP initiation relative risk of 1.19 (95% CI = 0.03 to 56.3; p = 0.93)) for implementation versus maintenance phase. Of those interviewed, 23 responded to the SUS and NPS questionnaire items, the mean SUS score of 82.0 (95% CI = 76.7 to 87.2) is considered an acceptable score, and the NPS of +61 is a score consistent with more respondents indicating they were promoters than detractors of the intervention.

**Table 3:** RE‐AIM Table

Type of Outcome	Specific Outcome	Value (%)
Reach	Unique attendings who launched the intervention at least once in the implementation phase.	19/68 (27.9%)
Effectiveness	Rate of BUP initiation in the ED (Implementation: Mar–Aug 2019).	22/332 (6.6%)
Adoption	Unique attendings who initiated BUP in the implementation phase.	16/68 (32.3%)
Implementation	Phenotype‐positive patients for whom the intervention was launched in the implementation phase.	28/332 (8.4%)
Maintenance	Rate of BUP initiation in the ED after training ended (Sep–Dec 2019).	11/208 (5.5%)

#### Qualitative Interview Feedback

As shown in Data Supplement S1, Appendix S2, categorization of qualitative feedback according to common themes revealed that the CDS decision support tools could be improved by tracking and displaying calculated scores (DSM‐5, COWS, etc.), on the main screen for easy reference and automatically highlighting the best care pathway based on these scores. Additional suggestions included making the decision support tools more visible and clarifying that they are optional and not required to launch a care pathway. Regarding EHR workflow, a common area of feedback was to increase clarity of what happens when a care pathway is launched. Other interview feedback focused on the need to decrease confusion of the referral process, particularly details of the referral timeline, coordination with external providers, required next steps, and how to explain this process to patients. Additional miscellaneous suggestions include clarification of which features are available to providers without an X‐waiver, increasing awareness of availability, continuation of one‐on‐one training to promote use, and addition of a feature alerting providers to possible OUD patients likely to benefit from BUP.

## Discussion

In this interrupted time series evaluating the preliminary efficacy of the EMBED intervention at a single site, implementation of a user‐centered CDS with a brief, just‐in‐time training was associated with close to a doubling in the BUP initiation rate in the ED for patients with OUD and receiving a prescription for naloxone at discharge. After adjusting for the temporal trend of physician waiver training, the increased rate of BUP initiation was no longer statistically significant. The primary outcome was lower in the maintenance phase compared to the implementation phase (Figure 1). Given the ED's significant role in caring for patients affected by the opioid epidemic, these results suggest that a user‐centered, well‐integrated CDS like EMBED is an efficacious approach to increase adoption of an effective treatment for OUD.

Despite the ED's potential to initiate treatment for a large number of patients with OUD, a cohort study conducted in Massachusetts indicates that this potential has yet to be realized. Among the 17,000 patients who had an ED visit for nonfatal opioid overdose between 2012 and 2014, only one in three received a MOUD in the 12‐month period following their overdose. Compared to no MOUD treatment, both methadone and BUP were associated with decreased all‐cause mortality and opioid‐related mortality.^11^ In light of these findings, successful initiation of BUP in the ED for the subjects in this study could have had a significant mortality benefit for these victims of nonfatal opioid overdose.^39^ Although the need for increased MOUD utilization in the ED is clear, the path to meeting that need is unfortunately not so simple.

A number of barriers currently limit the rate of BUP initiation in the ED. One such barrier directly addressed by the EMBED intervention is how the majority of emergency physicians feel unprepared to provide OUD care; a 2019 survey of physicians from two urban, academic EDs found that fewer than half of respondents felt prepared in several components of OUD emergency care.^40^ Only 39% of physicians self‐rated themselves as prepared to determine the level of care needed by an OUD patient, while 29% felt prepared to connect OUD patients with outpatient treatment. Of all surveyed components, emergency physicians felt least prepared to initiate BUP, with only 27% self‐reporting themselves as prepared. Specific features of the EMBED intervention are available to assist physicians feeling unprepared in each of these components, transforming what could be a time‐consuming and unfamiliar task into a simpler and more feasible one for all ED clinicians. The results of this interrupted time series study demonstrate the preliminary efficacy of this intervention on physician adoption rate of ED‐initiated BUP. This effect size could indeed be much larger as we did not collect data on other reasons for not starting treatment (e.g., patient readiness) given the pragmatic nature of the intervention.

Although 93% of emergency medicine faculty had an X‐waiver by the end of the preimplementation phase, the proportion of unique attendings who had adopted the practice of ED initiation of BUP was low (19.2%). In the implementation phase, the proportion of X‐waivered physicians increased slightly to 97%. However, during the implementation phase, we observed a close to a doubling in the proportion of unique attendings who had adopted the practice of ED initiation of BUP (19.2% vs. 32.3%, p = 0.53). Although this increase was not statistically significant, it seems more clinically significant and suggestive that the barrier of X‐waivered physicians actually adopting the practice of ED‐initiated BUP may require a user‐centered CDS with just‐in‐time training. Similarly, although not statistically significant, the proportion of unique attendings who initiated BUP after we began just‐in‐time training nearly doubled. Taking all of these findings together, we hypothesize that all three (X‐waiver training, user‐centered CDS, and just‐in‐time training) may be necessary and complementary to increase adoption of ED‐initiated BUP.

Creation of a CDS with these capabilities is the result of our choice to employ a user‐centered design process which involved identifying users' needs and incorporating their feedback in each phase of iterative prototype development.^23^ The value of user feedback and collaboration also motivated changes to the intervention. After it was discovered that intervention usage during the first half of the implementation phase was relatively low, one‐on‐one tutorials and qualitative data collection were added to the intervention to enhance its use and performance. More in‐depth qualitative analyses of implementation barriers are under way in four additional urban, academic EDs to determine additional ways to promote adoption of ED initiation of BUP.^41^

The decision to incorporate training as part of the intervention turned out to be one of the study's strengths. Although not statistically significant, increased rates of the primary outcome with one‐on‐one training component suggests that this feature of the intervention is necessary to increase user recognition of its presence and value. The training component also leverages the science of “diffusion of innovations,” as a way to promote intervention adoption via communication and sharing by local champions and among colleagues within a social system.^42^ To generate a tipping point for adoption through visibility and diffusion, this training feature will be encouraged as an intervention component in the upcoming trial to facilitate implementation at study sites. Despite the increase in BUP initiation associated with training, the sustainability of its effect is questionable. Data collected in the maintenance phase show a decrease in the rate of BUP initiation over the months following the conclusion of the training period. Examining the sustainability of such training as a long‐term solution versus a transient benefit as well as its impact on intervention scalability could be an area of future work.

Planning for the upcoming trial is also supported by the collection of qualitative data that resulted in a richer data set and a better understanding of barriers to adoption and possible solutions. Some of these findings include the need to increase awareness of the intervention, for the CDS to display calculated scores on the home screen, and to increase clarity of the referral process.

## Limitations

There are many barriers to adoption of ED initiation of BUP into routine emergency care. For example, the EMBED intervention does not address negative attitudes toward addiction, the inconvenience of obtaining an X‐waiver to prescribe BUP, the limited number of providers with an X‐waiver, and access and availability to MOUD in the community. This study was also conducted in a single ED with a high rate of X‐waivered physicians compared to nonacademic or rural EDs. Because physicians without an X‐waiver are limited in their ability to prescribe BUP for home induction, this could have had an impact on the primary outcome of rates of ED BUP initiation. Another weakness of our study's design is that it was neither randomized nor controlled, so causality between the intervention and outcomes could not be established. Given the urgency of the opioid crisis, additional temporal trends could have occurred that were not adjusted and remain as confounders. The increase in X‐waivered physicians, although identified and adjusted during analysis, is an example of one trend which alone could have impacted study results if overlooked. Finally, in an effort to avoid unintended consequences from a hard‐stop alert that triggered the CDS, not all attending physicians utilized the intervention during the implementation phase.^43^ Although the phenotype identified potentially eligible patients, the less interruptive nature of the intervention also meant that we did not collect data on actual presence or absence of OUD, withdrawal severity, and readiness for treatment. Therefore, the full potential and effect of the intervention on various outcomes may be underestimated or inaccurate.

Future research could explore alternative approaches to triggering the intervention in a nonobstructive manner and whether lack of a hard‐stop alert represents another barrier to physician adoption. Additional investigative efforts are necessary to explore the extent to which findings of the study are generalizable across different patient populations, health care systems, and EHR platforms. Similarly, the scalability of EMBED as a solution to increasing adoption of BUP initiation in the ED will require further investigation, particularly across different health care systems using different EHRs. The upcoming pragmatic trial to be launched in 20 EDs across five health care systems using different EHR vendors may provide answers to some of these remaining questions.

## Conclusion

This interrupted time series study demonstrates that implementation of the EMBED intervention in a single ED is feasible, acceptable, and estimated to be efficacious at increasing rates of buprenorphine initiation for the treatment of opioid use disorder in the setting of a temporal trend of increased physician X‐waiver training. The intervention was associated with a doubling of the buprenorphine initiation rate, emphasizing the importance of user‐centered health IT to change practice around evidence‐based medicine that may be slowly adopted. Our findings suggest that we should proceed with the larger effectiveness trial to explore whether these preliminary findings are significant and generalizable. Knowledge gained from the study will continue to inform the trial, particularly the finding of how one‐on‐one training is a necessary part of the intervention to increase and sustain adoption of clinical decision support.

## Supporting information


**Data Supplement S1.** Supplemental material.

**Data Supplement S2.** Supplemental material.
