# Supplementary material for: Interrupted Time Series of User‐centered Clinical Decision Support Implementation for Emergency Department–initiated Buprenorphine for Opioid Use Disorder
Source: Acad Emerg Med. 2020 May 19;27(8):753–63. doi: 10.1111/acem.14002 (PMC7496559; doi:10.1111/acem.14002)
Supplement: Supplementary file 1 — Data Supplement S1. Supplemental material. [file ACEM-27-753-s001.pdf]

Data Supplement S1. Supplemental material

Appendix S1. *Interview handout (SUS, NPS, open-ended questions)*

CLINICIAN APPLICATION USE FEED BACK SURVEY

Title: EMBED: Pragmatic trial of user-centered clinical decision support to implement EMergency department-initiated BuprenorphinE for opioid use Disorder

Principal Investigator: Edward R. Melnick, MD, MHS

Funding Source: National Institute on Drug Abuse

Date:_____________ Study ID:_____________ Provider Type:_________________

1. Have you previously utilized the EMBED application during actual patient care?

If answer **YES** to question #1:

2a. How was your experience using the EMBED application?

2b. Is there anything you would change to improve the EMBED application?

If answer **NO** to question #1:

3a. If you have not used the EMBED application yet with a patient, why not? *(Note: see separate document "Anticipated Responses and How to Support" as needed)*

3b. Is there anything you would change in this application to increase the likelihood that you would utilize the EMBED application during actual patient care?

System Usability Scale

Please select the answer that best expresses how you feel about each statement after using the EMBED application today.

	Strongly Disagree	Somewhat Disagree	Neutral	Somewhat Agree	Strongly Agree
1. I think I would like to use this tool frequently.	☐	☐	☐	☐	☐
2. I found the tool unnecessarily complex.	☐	☐	☐	☐	☐
3. I thought the tool was easy to use.	☐	☐	☐	☐	☐
4. I think that I would need the support of a technical person to be able to use this system.	☐	☐	☐	☐	☐
5. I found the various functions in this tool were well integrated.	☐	☐	☐	☐	☐
6. I thought there was too much inconsistency in this tool.	☐	☐	☐	☐	☐
7. I would imagine that most people would learn to use this tool very quickly.	☐	☐	☐	☐	☐
8. I found the tool very cumbersome to use.	☐	☐	☐	☐	☐
9. I felt very confident using the tool.	☐	☐	☐	☐	☐

10.	I needed to learn a lot of things before I could get going with this tool.	☐	☐	☐	☐	☐

Net Promoter Score

“On a scale of 0-10, how likely is it that you would recommend EMBED to a colleague?”

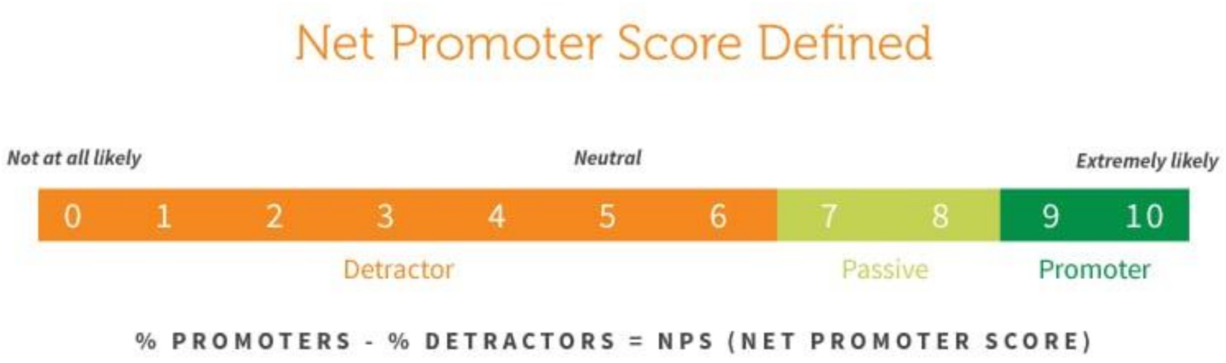

Appendix S2. *Clinician Feedback*

Topic	Barrier	Provider feedback for addressing barrier
Decision Support	Calculated scores not displayed on main screen	-Application should display calculated score on main screen for reference
	Best care pathway not indicated	-Systematic narrowing of remaining care pathways based on decision support tool calculations<br><br>-Best care pathway for patient should be highlighted
	Design and interface	-Increase visibility of decision support column and tools<br><br>-Make it clear that steps flow in vertical (not horizontal) direction<br><br>-Simplify/shorten text of COWS assessment<br><br>-Clarify that decision support tools are optional and not required to launch a pathway<br><br>-Decision support tool for readiness motivation should include a message/note stating that there is nothing to click
Automated EHR Workflow	Uncertainty of what happens when a care pathway is launched	-Application should display a summary of automated orders sent and actions taken following each launch of a care pathway
Referral Process	Referral process timeline unclear	-Application should specify or approximate date of referral visit<br>-Application should provide information on what happens next<br>-Applications should display length of time between discharge and referral visit so BUP can be prescribed for home if necessary
	Unclear how to explain referral process to patients	-Application should provide instructions to give patient explaining referral process and next steps (e.g. does the referral center contact the patient following discharge?)

X-Waiver	Confusion of which CDS features are available to unwaivered clinicians and how to proceed without a waiver	-Application should provide a list of waivered ED clinicians currently working who can sign a BUP prescription on behalf of an unwaivered clinician
Unfamiliarity	Unawareness of intervention availability	-Change CDS icon name from "EMBED" to something more obvious like "Buprenorphine"
	Lack of clinician understanding on how to use CDS	-One-on-one training was beneficial and should be available from the beginning to help introduce the CDS and increase use
Other	Mobility of computers is limited	-More computers on wheels should be available for use of CDS at patient bedside or in crowded hallway<br><br>-Creation of a mobile application for use at patient bedside
	CDS does not automatically identify patients with OUD	-Add a feature that flags patients likely to have OUD and sends an alert or reminder to care team
